# Notes and News

**Published:** 1894-07-28

**Authors:** 


### Notes and News.

Collected and Communicated.

MEETINGS OF THE WEEK.

Hospital for Consumption and Diseases of the Chest, Brompton, Quarterly Court in the Board Room, August 2nd, at 4.45 p.m.

The sum of £800 has been the brilliant result of Madame Sarah Bernhardt's reception at the Grafton Galleries in aid of the French Hospital.

A home and hospital for aged men is to be established at Cork, according to the will of Mr. Honan of that city, who has left £25,000 for the purpose. The home is for Roman Catholics of good character.

Ladies in America seem confident that their services are required in the field of medicine, and each year sees a larger number of fair graduates from the various medical schools. No fewer than fifty women doctors received their degrees recently at the Women's Medical College of Pennsylvania.

In recognition of the services of the late Dr. Arthur Hill Hassall, whose name will be remembered in connection with various questions of public health, Lord Rosebery has granted Mrs. Hassall a pension of £50 per annum from the Civil List. Dr. Hassall did good work in the detection of adulterations, and was the first to use the microscope on a large scale for this purpose. He was for many years physician and senior physician to the Royal Free Hospital, and was the founder of the Royal National Hospital for Consumption, Ventnor. In the large hall of this institution a memorial, a portrait of Dr. Hassall, as founder, is about to be placed.

Rather an amusing war has been fought in Texas between the State Medical Society and a newspaper. The newspaper stated that a member of the society, speaking at a meeting, advocated a system of suppression of patent medicines, not for the patients' but for the doctors' sake, as by written prescriptions both druggist and doctors made a larger profit. Further, the paper quoted instances of patent medicines being used as ingredients in written prescriptions by some doctors. The society called a meeting, and passed several resolutions to the effect that the newspaper article was false and libellous, and so the matter was dismissed.

The Hospital Saturday Fund collection has so far reached the sum of £9,568, of which £5,238 comes from the workshop and £4,330 from the street collection.

The East London Hospital for Children, Shadwell, is the richer for several donations from the City companies—the Goldsmiths' Company having sent £50, and the worshipful companies of Cordwainers and Curriers donations of £10 and £5 respectively.

Everywhere reformers are busying their brains to discover a solution of the drink question. Quite a novel scheme is to be tried in Massachusetts. The method may be called a homœopathic one. A society is to be formed to supply alcoholic beverages at the lowest possible cost and of the best quality. The idea is that the huge saloon of the society will gradually attract all customers from the private vendors, who, thus ruined, will have to retire from their trade. The day won, the saloon will be closed, but will immediately reopen upon any rival again appearing in the field. There is a great deal of enterprise in the scheme, but whether the trade and people of the locality will consent to be manipulated in such a manner is quite another thing.

At this time of the year all classes are arranging for such holiday making as circumstances permit. None need this holiday more than the workers in London factories. The Factory Girls' Country Holiday Society make an especial appeal now to enable them to send as many tired workers into the country as possible. The holiday makers are drafted into various country homes carefully chosen by the country organisers, and remain, as a rule, for a fortnight. Last year the society was able to provide for 463 girls, who themselves contributed over £78 to the funds. A glance at the report, to be procured from the hon. secretary, Miss Canney, St. Peter's Rectory, Saffron Hill, E.C., will convince the reader how genuine is the benefit and enjoyment which a moderate donation can secure to those who have few opportunities of securing either health or innocent pleasure such as a visit to the country affords.

Whilst Ireland is establishing her National Hospital for Consumption, in addition to existing establishments for phthisis, the scheme for the first hospital of the kind in Scotland has been left to the initiation of a private individual. Mr. William Quarrier, a well-known and active philanthropist, is about to enter into the undertaking of providing the first establishment of the kind, on land adjoining his Orphan Homes at Bridge of Weir, near Glasgow. It would appear that Mr. Quarrier intends to follow the plan of the separate system, as at Ventnor. He has been provided by a friend with £7,000 to build his first "Home," to accommodate 30 patients. He wishes eventually to erect six houses, the whole scheme to cost £50,000. So far, £10,000 are forthcoming, but it is to be expected that the Scotch nation, once awake to so great a want as a consumption hospital in a country where the disease is so prevalent, will come forward and reward Mr. Quarrier for his public-spirited and humane intentions by providing him with even more than the necessary means to carry them into effect.

